# Novel lincRNA Discovery and Tissue-Specific Gene Expression across 30 Normal Human Tissues

**DOI:** 10.3390/genes12050614

**Published:** 2021-04-21

**Authors:** Xianfeng Chen, Zhifu Sun

**Affiliations:** Division of Computational Biology, Department of Quantitative Health Sciences, Mayo Clinic, Rochester, MN 55905, USA; Chen.Xianfeng@mayo.edu

**Keywords:** long intergenic non-coding RNA, lincRNA, GTEx, RNA sequencing, tissue-specific gene expression, human normal tissue, bipolar disease, GWAS SNPs

## Abstract

Long non-coding RNAs (lncRNAs) are a large class of gene transcripts that do not code proteins; however, their functions are largely unknown and many new lncRNAs are yet to be discovered. Taking advantage of our previously developed, super-fast, novel lncRNA discovery pipeline, UClncR, and rich resources of GTEx RNA-seq data, we performed systematic novel lincRNA discovery for over 8000 samples across 30 tissue types. We conducted novel detection for each major tissue type first and then consolidated the novel discoveries from all tissue types. These novel lincRNs were profiled and analyzed along with known genes to identify tissue-specific genes in 30 major human tissue types. Thirteen sub-brain regions were also analyzed in a similar manner. Our analysis revealed thousands to tens of thousands of novel lincRNAs for each tissue type. These lincRNAs could define each tissue type’s identity and demonstrated their reliability and tissue-specific expression. Tissue-specific genes were identified for each major tissue type and sub-brain region. The tissue-specific genes clearly defined each respective tissue’s unique function and could be used to expand the interpretation of non-coding SNPs from genome-wide association (GWAS) studies.

## 1. Introduction

Long non-coding RNAs (lncRNAs) are a large class of gene transcripts that do not code proteins (vs. mRNAs that translate to proteins). Reports suggest up to 68% of transcribed genes could be from lncRNAs [1,2]. Based on their location with respect to protein-coding genes, there are at least five categories of lncRNAs: antisense lncRNAs, which intersect any exon of a protein-coding locus but on the opposite strand; long intergenic non-coding RNA (lincRNAs), which are coded in an intergenic region with a length > 200 bp; sense overlapping lncRNAs, which contain a coding gene within an intron on the same strand; sense intronic lincRNAs, which sit in introns of a coding gene but do not overlap any exons; and processed transcripts, which do not contain an open reading frame [3,4]. LncRNAs interact with DNA, mRNA, protein, or miRNA and play an important role in gene expression regulation and processing at multiple levels [5,6]. They are found to be involved in multiple diseases such as cancer [3,7,8], autoimmune diseases [9,10], and neurological disorders [11]. Traditional RNA sequencing analysis is mostly based on known gene annotation and lncRNAs that are not annotated would not show up in downstream analysis, which could potentially miss a lot of useful information. However, identifying uncharacterized lncRNAs is computationally challenging for most researchers. We previously developed integrated and super-fast novel lncRNA discovery pipeline UClncR [12], which is fully automated starting from fastq files or aligned bam files to novel lncRNA reports across samples. Depending on RNA-seq library protocols, UClncR can detect all lncRNAs as described above for stranded RNA-seq or lincRNAs only for unstranded RNA-seq. This is because stranded RNA-seq retains coding strand information in sequenced reads so that overlapping lncRNAs with protein-coding or other genes can be distinguished using the strand information. On the other hand, unstranded RNA-seq does not have this information (reads equally mapped to both forward and reverse strands) so that only lincRNAs without any overlap with other genes can be reliably detected.

The Genotype-Tissue Expression (GTEx) project provides a large resource for the research community to study human gene expression and regulation and its relationship with genetic variation on tens of thousands of samples across over 30 major human tissue types [13,14]. Gene expression data for this project are obtained from unstranded RNA-seq with GENCODE annotation (v26 for release V8 and v19 for release V7). Many papers have been published using GTEx data in recent years; however, little effort has been made to detect novel lncRNAs, and information about tissue-specific gene expression across different tissue types is limited, although tissue-specific expression measured as tissue specificity score in identifying drug target context was explored [15].

Taking advantage of the rich RNA-seq data from GTEx and our fast UClncR pipeline, we performed systematic novel lincRNA detection and characterization of more than 8 K samples from 30 human tissue types. We conducted novel detection for each major tissue type first and then consolidated the novel discoveries from all tissue types. These novel lincRNs were then added to known genes in tissue-specific gene expression analysis to identify tissue-specific genes for each tissue type. We finally used brain tissue-specific gene expression to illustrate its potential utility in enhanced interpretation of GWAS-associated SNPs in bipolar disease.

## 2. Materials and Methods

### 2.1. GTEx RNA-seq Samples, Pre-Processing, and Novel lincRNA Detection

Raw data for 8584 samples from GTEx (release V6) were downloaded from dbGAP and converted to fastq format. The raw data for each sample were processed by our updated MAPRseq pipeline (V3), where reads were mapped to HG38 by STAR [16] (Supplementary Figure S1). Cell line samples (bone marrow, transformed fibroblast, and transformed lymphocytes) and samples with less than 35 million reads were dropped. Mapped reads with mapping quality scores less than 10 were filtered out (only uniquely mapped reads were kept). The final dataset had 8046 samples (sequence depths from 35 to 470 million reads, with a median of 86 million) from 30 major tissue types. The aligned files (BAM) were then processed by UClncR [12] for novel lincRNA detection for each tissue type separately, where GTFs from each sample were merged using StringTie [17] and gene expression quantification was performed using this merged GTF along with known gene GTFs (Ensembl release 93). The GTFs from each tissue type were then further merged to create a meta-meta GTF for all tissues and samples, which was used along with known gene GTFs to create a unified gene expression matrix by featureCounts [18] for all samples together. As RNA-seq from GTEx was from an unstranded protocol, only novel lincRNAs were detected in this study. Further, only more reliable multi-exon novel lncRNAs were used for additional analysis with known genes for tissue-specific gene expression.

### 2.2. Tissue-Specific Gene Identification

**Figure 1:**
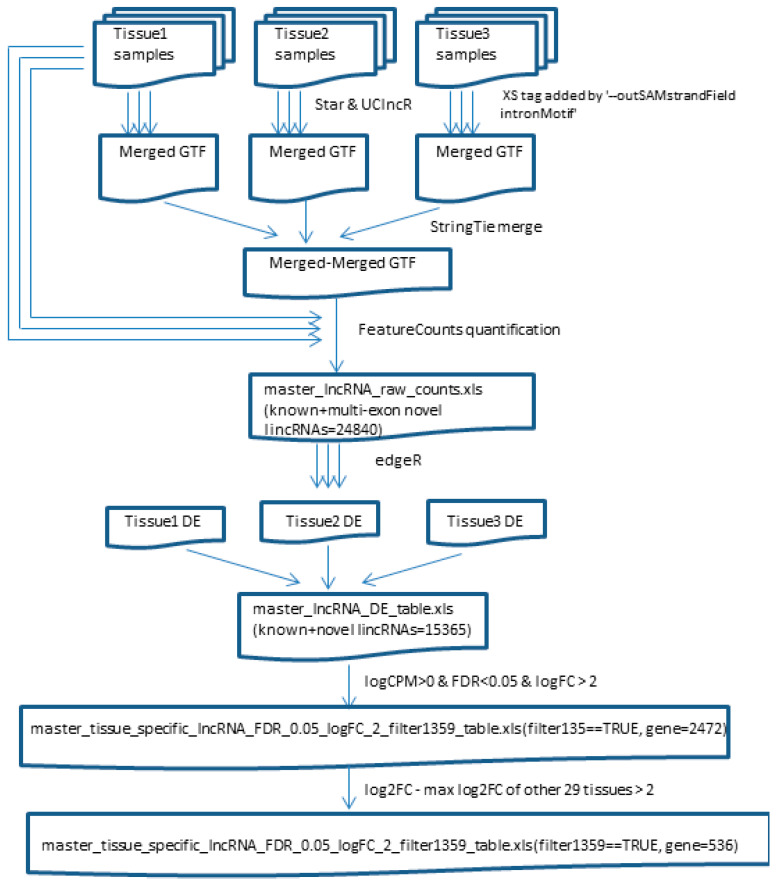
Workflow of novel lincRNA detection and tissue-specific gene expression.

To obtain genes that were specifically expressed in each tissue type, we compared each tissue type to all other tissue types for both known and newly detected lincRNAs using edgeR [19]. Because of the variable sample sizes from different tissue types, we randomly selected 100 samples for those with more than 100 samples. Genes that had average log2 count per million (CPM) > 0, false discovery rate (FDR) < 0.05, and log2 fold change greater than >2 (only upregulated in the tissue of interest) were defined as the first-tier tissue-specific genes. To further select more specific genes for each tissue type, the log2 fold change greater than 2-fold above the maximum log2 fold change of another 29 tissue types was also used. Further details of the analyses are presented in Figure 1.

We also performed a similar analysis for brain tissues as the brain contains 13 sub-brain regions or parts. Because of the high similarities among different brain regions, tissue-specific genes were defined using less stringent criteria of log2 CPM > 0, FDR < 0.05, and log2 fold > 0.585 (1.5 fold).

### 2.3. Enrichment of GWAS Significant SNPs of Bipolar in Brain-Specific Genes

To illustrate the potential use of novel lincRNA and tissue-specific genes in SNP interpretation, we collected 19,861 bipolar-associated SNPs from GWASDB [20], the GWAS catalog [21] (https://www.ebi.ac.uk/gwas/; accessed on 5 March 2019), and a recent literature review [22] and overlapped these with the genes used for the tissue-specific gene expression. The number of GWAS hits in brain-specific vs. non-brain-specific genes was counted for enrichment analysis by Chi square test.

## 3. Results

### 3.1. UClncR Detected a Significant Number of Novel lincRNAs from Each Tissue Type

**Figure 2:**
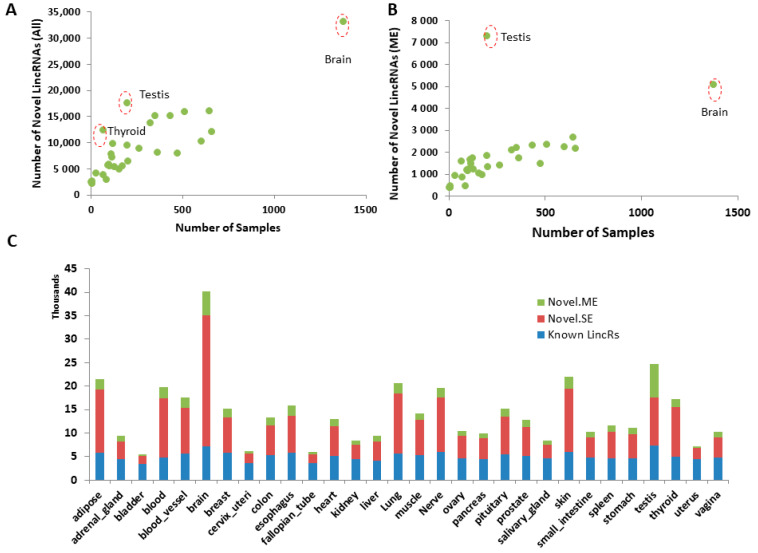
Statistics of novel lincRNAs in each tissue. (**A**) Number of samples vs. number of detected novel lincRNAs (both single and multi-exon lincRNAs). (**B**) Number of samples vs. number of detected novel multi-exon lincRNAs. (**C**) Relative numbers of known, novel single-exon, and novel multi-exon lincRNAs in each tissue type. Novel.ME—novel multi-exon lincRNAs, Novel.SE—novel single-exon lincRNAs, Known lincRs—Known lincRNAs in Ensembl annotation.

We performed novel lincRNA detection for each tissue type separately and the number of novel lincRNAs detected from each generally increased with the number of samples in a particular tissue type, ranging from 2140 in bladder tissues to 33,017 in brain tissues (Figure 2A). However, some tissue types, such as testis and thyroid, appeared to have a disproportionally higher number of novel lincRNAs relative to the number of samples in the analysis. This is particularly true for novel lincRNAs with multiple exons. For example, testis only had 201 samples but it had the highest number of novel multi-exon lincRNAs detected (7282 vs. 5072 from brain, which had 1378 samples, the highest of all) in all tissue types (Figure 2B).

For expressed lincRNAs (read count > 5 in at least one sample), the novel single-exon lincRNAs doubled or tripled the number of known lincRNAs in each tissue type (Figure 2C). The novel multi-exon lincRNAs accounted for 10 to 100% of known lincRNAs. Altogether, novel lincRNAs doubled or tripled the number of known lincRNAs in each tissue type. Consolidating all novel detections from all tissues, 17,427 potential novel lincRNAs were detected, which cover 402.4 Mb of the human genome (vs. 236.3 Mb of the known lincRNAs), which significantly expanded the coding region of the genome (known lincRNA accounting for 7.9% and novel lincRNA accounting for 13.4% of the genome).

### 3.2. Novel lincRNAs from Each Tissue Type Define Their Tissue Type

**Figure 3:**
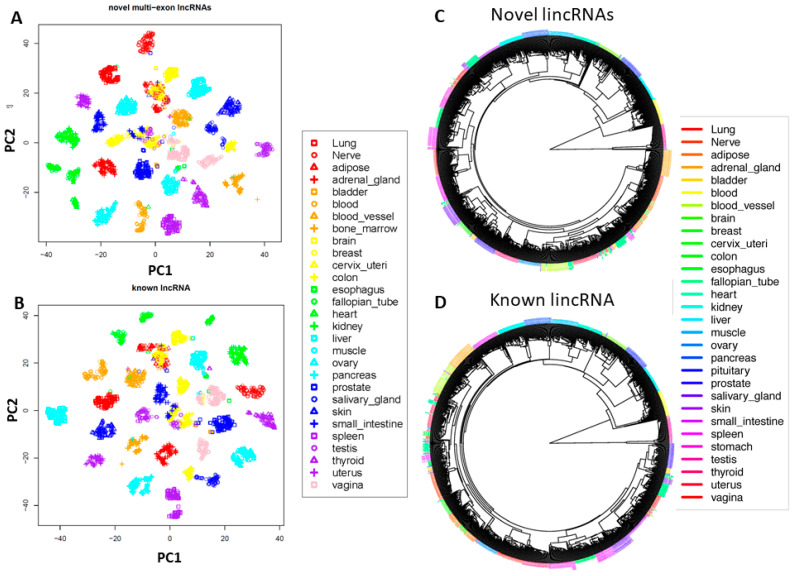
Novel multi-exon lincRNAs define tissue types. (**A**) t-distributed stochastic neighbor embedding (t-SNE) unsupervised clustering for all samples using novel multi-exon lincRNAs. Each tissue type forms a distinct cluster. (**B**) t-SNE unsupervised clustering for all samples using known lincRNAs. (**C**) Unsupervised clustering in circular format to show the relative relationship among samples using novel multi-exon lincRNAs. (**D**) Unsupervised clustering in circular format to show the relative relationship among samples using known lincRNAs.

We conducted unsupervised dimension reduction through machine learning for both known and novel lincRNAs with multiple exons as they are generally more reliable with exon junction alignment support compared to single-exon novel lincRNAs. Like known lincRNAs, the newly detected lincRNAs could separate different samples of tissue origin into distinct clusters (Figure 3A,B), suggesting their tissue specific expression and our pipeline’s reliability. Using unsupervised clustering, the novel lincRNAs were also able to reveal the relatedness of different tissue types. For example, tissues from the gastrointestinal tract (stomach, small intestine, and colon) were clustered closely together and neurological tissues (brain and pituitary) were more similar (Figure 3C,D).

### 3.3. Identification of Tissue-Specific Genes (lincRNAs and Protein Coding Genes)

**Figure 4:**
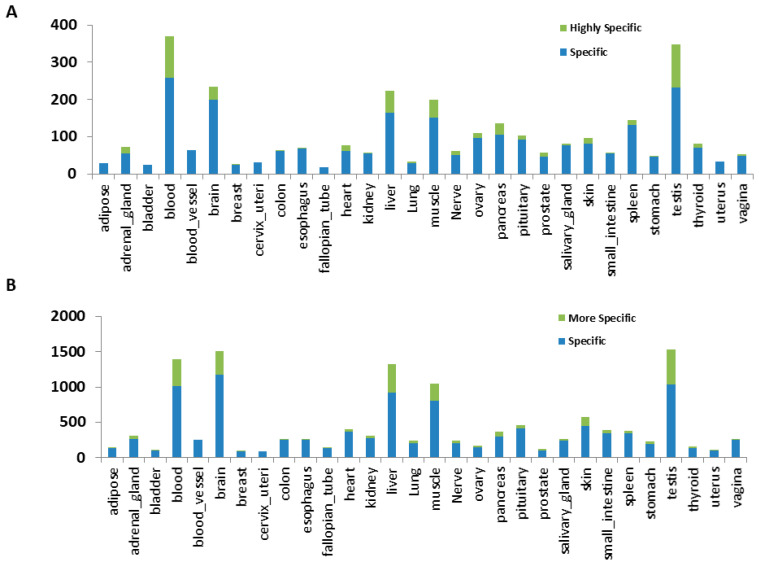
Number of tissue-specific lincRNAs and protein-coding genes. (**A**) lincRNAs. (**B**) Protein-coding genes.

With the unprecedented diverse tissue types and numbers of samples, it is very useful to define tissue-specific genes that express more specifically or uniquely in a particular tissue type, which not only help to define a tissue type as a marker gene but also elucidate each tissue’s unique functions. For this, we conducted one vs. all remaining analysis for each tissue type. At FDR < 0.05, average read count per million greater than 1 and log2 fold change greater than 2 (4-fold, upregulated only), variable numbers, ranging from 18 to 258, of tissue-specific lincRNAs (both know and novel) were identified for each tissue type (Figure 4A, blue bar). As comparison, the numbers of tissue-specific protein-coding genes were also detected (Figure 4B, blue bar). The numbers of tissue-specific lincRNAs in each tissue type were proportionally similar to the numbers of tissue-specific protein-coding genes. Blood, brain, liver, muscle, and testis were the tissues with the highest numbers of tissue-specific lincRNAs or protein-coding genes. Using a more stringent fold change filter (more than 2 fold of the highest fold change of all other tissues), we identified more specific lincRNAs or protein-coding genes in most of the tissues (Figure 4A,B, green bar and marked as “highly specific”), although some tissues, such as the ones with dominant smooth muscle tissue (bladder, blood vessel, cervix, and uterus), had none. Again, we found that blood, brain, liver, muscle, and testis had a higher number of highly tissue-specific genes. Overall, 26.02% of genes were tissue-specifically expressed in at least one of 30 tissues. While 38.17% protein-coding genes were tissue-specific in at least one of the tissues, around 11.08% lincRNAs were tissue-specifically expressed (known 7.90% and novel 12.02%). Genes specific to at least one tissue are provided in Supplementary Table S1.

To illustrate whether these highly tissue-specific genes/lincRNAs perform specific functions, we used testis as an example as it had the highest number of “highly specific” lincRNAs (115 lincRNAs) and protein-coding genes (497 genes) among all the tissues. Among the lincRNAs, 84 were newly detected (vs. 31 known). Pathway enrichment analysis for the protein-coding and lincRNA-associated protein-coding genes (the closest gene from each lincRNA) showed significant enrichment of cell cycle (*p* value = 0.00005) and oocyte meiosis (*p* value = 0.01). The top five upregulated genes (by fold change) were CETN1, PRR30, LELP1, ACTRT2, and HMGB4. The top five lincRNAs were three known (AC092447.8, LINC00917, and LINC01921) and two newly detected (chr7:57216135-57219296 and chr17:7428378-7433068). The brain is another specialized organ with many tissue-specific expressed genes. The top enriched pathways were nicotine addiction, GABAergic synapse, synaptic vesicle cycle, and morphine addition (all with enrichment *p* values less than 4.7 × 10^−13^), the pathways well known for brain functions. The top three specific protein-coding genes are OPALIN, AVP, and CACNG3, while the top three lincRNAs are LINC00599, AL031056.1, and MSTRG.27248 (novel one at chr18:66622040-66712235, upstream of CDH19).

### 3.4. Tissue-Specific Genes in Different Parts of the Brain

**Figure 5:**
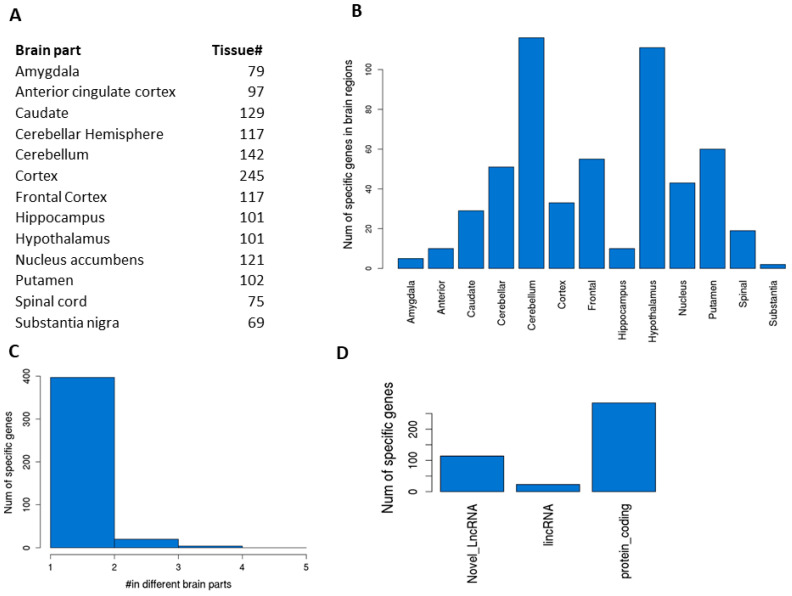
Brain subregion-specific genes. (**A**) Number of samples in each sub-brain region. (**B**) Number of specific genes in each subregion of the brain. (**C**) Distribution of specific genes across sub-brain regions. Vast majority are unique to each brain part while some are found to be unique to 2–4 different brain parts, which generally suggests that these regions have more similarity in function or anatomic location. (**D**) Numbers of sub-brain specific genes across gene category.

The brain is the most complex organ, with multiple subregions or parts performing different functions. Each subregion has different types of cells or proportions of the same cells. Understanding their gene expression differences would help to understand their unique functions. For 13 subregions of the brain in GTEx (Figure 5A), we conducted similar vs. all remaining differential gene expression analysis for tissue-specific genes (protein-coding, known lincRNAs, and novel lincRNAs only). Because of their higher similarity, as expected, we saw only a few genes that met the stringent criteria defined for the main organ comparisons; thus, we reduced the fold change to 1.5 (log2 0.585, with other criteria remaining the same). This analysis led to 2 to 116 sub-brain-specific genes (Figure 5B and Supplementary Table S2). The cerebellum and hypothalamus were the top two regions with the highest number of specific genes (116 and 111, respectively). The vast majority of these sub-brain-specific genes (326 genes) were very unique as they only appeared once in one of the sub-brain regions (Figure 5C) while some other genes (95 genes) were shared among different sub-brain regions, suggesting that these regions were more similar in terms of function or anatomic location. For example, GBP5 was detected to be specific to the cerebellar hemisphere, cortex, frontal cortex, and hippocampus and GAL to the cerebellum, frontal cortex, hypothalamus, and putamen. Among the unique sub-brain-specific genes, 284 are protein-coding, 23 are known lincRNAs, and 114 are newly detected novel lincRNAs (Figure 5D).

**Figure 6:**
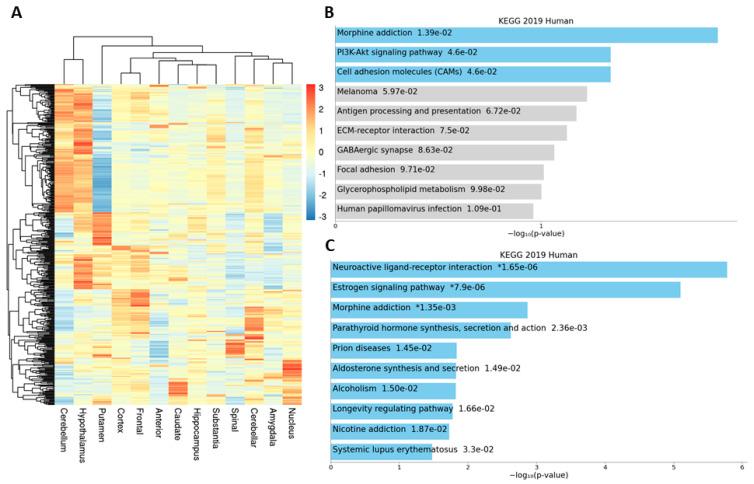
Sub-brain-specific genes. (**A**) Heatmap of 410 sub-brain-specific genes. Each row is a gene whose expression is averaged for each sub-brain region. (**B**) Enriched pathways for cerebellum-specific genes. (**C**) Enriched pathways for hypothalamus.

We explored the relationship among sub-brain regions using the heatmap for the 410 genes (gene expression was averaged across samples for each sub-brain) (Figure 6A). Pathway enrichment analysis for cerebellum-specific genes showed the significant pathway enrichment of morphine addition, PI3K-Akt signaling, and cell adhesion molecules (Figure 6B), while tissue-specific genes for the hypothalamus were enriched in neuroactive ligand reception interaction, estrogen signaling, and morphine addition as the top three (Figure 6C).

### 3.5. Bipolar GWAS-Associated SNPs in Brain-Specific Genes

**Figure 7:**
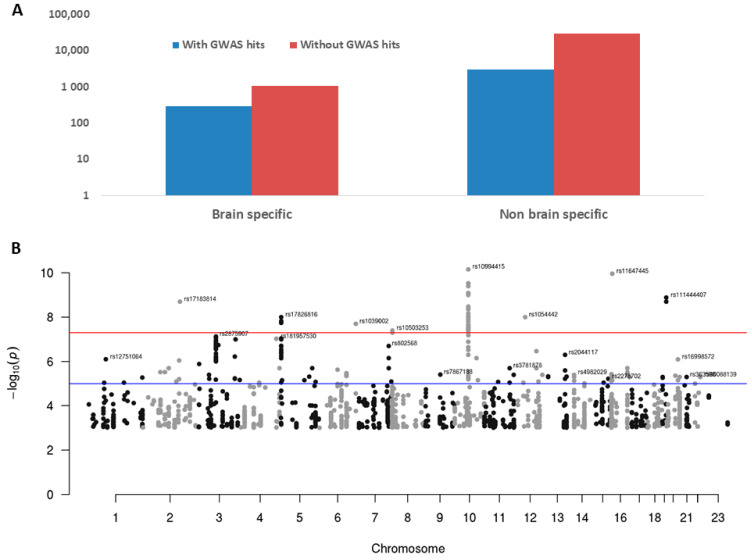
Bipolar GWAS hits in brain-specific genes. (**A**) GWAS hits in brain-specific genes vs. non-brain-specific genes (21.42% vs. 9.45%). Note: Y axis is log10 scaled to show the low numbers of brain-specific genes; however, the relative heights of the bars may distort their true absolute numbers. (**B**) Manhattan plot for GWAS hits mapped to brain-specific genes. *Y*-axis is −log10 (*p* value) from GWAS studies. Red line for genome-wide significant level (*p* value 5 × 10^−8^) and blue line is for suggestive *p* value of 1 × 10^−5^.

Genome-wide association studies (GWAS) have identified tens of thousands of genomic variants that are associated with a variety of human diseases or traits [23,24]. However, over 90% of them are located in the non-coding region of the genome [24], which makes interpretation difficult. We hypothesize that many of these non-coding variants could be in the region of regulatory elements such as lncRNAs and tissue-specific genes may better explain the associated variants with a particular disease. To test this, we downloaded bipolar-associated SNPs from three sources (see Materials and Methods section for details) and overlapped them with brain-specific genes detected in our analysis. Among the 19,861 GWAS hits, 10,899 (54.88%) were mapped to genic regions, of which 2034 were mapped to newly detected novel lincRNAs, accounting for around 20% of “interpretable” SNPs that would be missed if only known genes were considered. We further checked the distribution of these mapped SNPs in brain-specific and non-brain-specific genes and found that the brain-specific genes (at least one SNP mapped) had much higher proportions with mapped significant GWAS SNPs than the non-brain-specific genes (21.42% vs. 9.45%, chi square *p* value < 2.2 × 10^−16^, Figure 7A). A total of 1662 GWAS significant SNPs were mapped to brain-specific protein-coding genes, nine to known lincRNAs, and 23 to novel lincRNAs (Supplementary Table S3, Figure 7B). Of note, among the top 30 SNPs reported recently in a large-scale GWAS study, five of them were mapped to five brain-specific genes (ADCY2, ANK3, GRIN2A, NCAN, SCN2A) [22].

## 4. Discussion

The GTEx project provides rich resources for the research community to identify the functional impacts of genomic variants on phenotypes in a tissue-specific manner, particularly for gene expression from RNA-seq data [14,25]. Analyses so far are mostly based on known gene annotation, which could potentially miss genes not characterized before. We hypothesized that there could be many uncharacterized novel lncRNAs in the data that could be potentially useful for our understanding of gene regulation or interpretation of SNP variation in the non-coding regions. Using our previously developed superfast UClncR [12], we were able to process over 8000 samples efficiently and many novel lincRNAs were identified for each tissue type. These novel lincRNAs were able to uniquely define each tissue type. We next identified tissue-specific genes for each major tissue type and each sub-brain region. These tissue-specific genes have multiple potential uses. They can explain the tissue’s unique functions; they can be used as tissue-specific markers; genomic variants affect gene regulation and uniquely expressed genes or functions in a tissue may contribute to the preference of a disease in a particular organ; and they may be also highly valuable for drug selection [15].

The number of novel lincRNA discoveries is generally correlated with the number of samples in the analysis as lincRNAs could be expressed specifically to some individuals at particular physiological conditions (age, sex, environmental stimulus, etc.). The unexpected higher number of novel lincRNAs in testis may contribute to its uniqueness and less likely inclusion in previous studies in novel gene discovery. Our tissue-specific expression and GWAS hit enrichment analysis did not include single-exon novel lincRNAs as they are less reliable compared to multi-exon novel lincRNAs (without exon–exon junction read support). They could also be the result of fragmented transcripts during the multi-gtf merging process in the analysis. A closer examination of their suitability in interpretative analysis is needed in the future.

In our tissue-specific gene expression analysis, some tissues did not show highly tissue-specific genes or only showed a few. This is likely the result of tissue similarity among some of them, along with our stringent criteria—for example, uterus and “cervix_uteri”. It might be appropriate to combine some of the tissues or redefine the criteria to find tissue-specific genes for these tissues.

Our initial application of tissue-specific genes demonstrated the significant enrichment of bipolar GWAS hits in the brain-specific genes. This opens the door to expanding the interpretation of any disease-associated GWAS SNPs. Further work on how these SNPs affect disease development will be highly relevant and interesting.

## 5. Conclusions

In conclusion, we have discovered many novel lincRNAs in human normal tissues and defined tissue-specific genes for each tissue type. These data deepen our understanding of the transcription landscape and expand the interpretability of non-coding variants from GWAS studies.

## Data Availability

The data used for this search were downloaded from the GTEx project, which is publicly available at https://www.gtexportal.org/home/ (accessed and downloaded on 20 May 2018).

## Supplementary Materials

The following are available online at https://www.mdpi.com/article/10.3390/genes12050614/s1, Supplemental Table S1: Tissue-specific genes in 30 major GTEx tissues, Supplemental Table S2: Sub-brain-specific genes, Supplemental Table S3: GWAS significant SNPs with bipolar disease mapped to brain-specific genes, Supplementary Figure S1: Flow diagram of RNA-seq preprocessing pipeline MAPRseq (V3).
