# Supplementary figures and images for: Novel lincRNA Discovery and Tissue-Specific Gene Expression across 30 Normal Human Tissues
Source: Genes (Basel). 2021 Apr 21;12(5):614. doi: 10.3390/genes12050614 (PMC8143134; doi:10.3390/genes12050614)

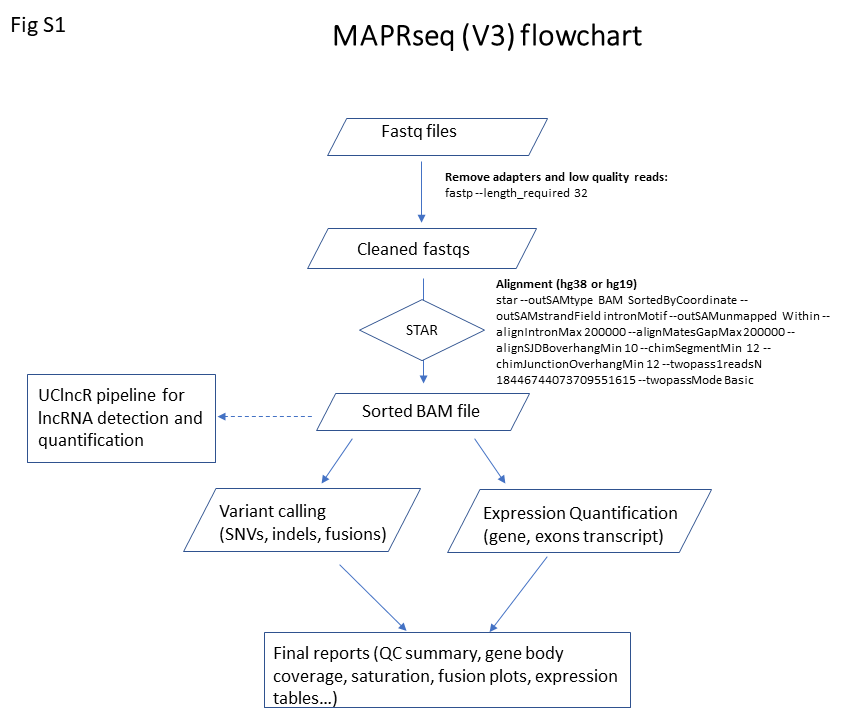

Supplement: Supplementary file 1 [file genes-12-00614-s001.zip › Supplementary Figures 1.tif]
